# Microhabitat Selectivity of Mites (Acari) in a Natural Lowland Beech Forest (*Melico*-*Fagetum*) in Wronie Reserve (Poland)

**DOI:** 10.3390/insects16040364

**Published:** 2025-04-01

**Authors:** Radomir Graczyk, Sławomir Kaczmarek, Tomasz Marquardt, Krzysztof Gęsiński, Dariusz J. Gwiazdowicz

**Affiliations:** 1 Department of Biology and Animal Environment, Bydgoszcz University of Science and Technology, Mazowiecka 28, 85-084 Bydgoszcz, Poland; graczyk@pbs.edu.pl; 2 Department of Evolutionary Biology, Faculty of Biological Sciences, Kazimierz Wielki University, Ossolińskich 12, 85-094 Bydgoszcz, Poland; slawkacz@ukw.edu.pl (S.K.); tmarq@ukw.edu.pl (T.M.); 3 Department of Microbiology and Plant Ecology, Bydgoszcz University of Science and Technology, Al. Prof. S. Kaliskiego 7, 85-796 Bydgoszcz, Poland; gesinski@pbs.edu.pl; 4 Department of Forest Entomology and Pathology, Poznań University of Life Sciences, Wojska Polskiego 71c, 60-625 Poznań, Poland

**Keywords:** Oribatida, Mesostigmata, biodiversity, bioindicators, ecology, *Fagus*

## Abstract

The European beech (*Fagus sylvatica* L.) is a tree species common throughout Europe, with the eastern boundary of its range extending across Poland. Samples were collected in several microhabitats of beech stands (e.g., leaf litter, rotting wood, moss growing on tree stumps) in the Wronie Forest Reserve. The aim of this study was to determine the effect of microhabitat conditions found in beech forests on the diversity of mite assemblages and their species richness. The collected samples comprised a total of 144 identified species (78 species of Oribatida, 66 species of Mesostigmata), represented by 74,433 mite individuals (71,124 Oribatida and 3309 Mesostigmata). All the analyzed microhabitats varied in terms of their mite assemblages. The highest number of species was identified in moss on beech stumps (72 species—53 Oribatida and 19 Mesostigmata) and in samples collected from beech litter (68 species—48 Oribatida and 20 Mesostigmata). The most numerously represented species in the analyzed material was *Parachipteria willmanni*, which was classified to superdominants in moss on beech stumps and moss on beech trunks (0.5 and 2.0 m), which were the most similar microhabitats. In contrast, mite assemblages in rotting wood and marsh litter differed greatly.

## Simple Summary

The European beech (*Fagus sylvatica* L.) is a tree species common throughout Europe, with the eastern boundary of its range extending across Poland. Material for the analyses was collected from several microhabitats of beech stands in the Wronie Forest Reserve. The acarofauna, and in particular Oribatida and Mesostigmata, inhabiting the microhabitats of beech stands has not been thoroughly explored so far. The aim of this study was to determine the effect of microhabitat conditions found in beech forests on the diversity of mite assemblages and their species richness. In the examined material, 144 taxa were recorded (78 species of Oribatida, 66 species of Mesostigmata). All the analyzed microhabitats varied in terms of their mite assemblages. The highest number of species was identified in moss on beech stumps (72 species) and from beech litter (68 species). The most numerously represented species in the analyzed material was *Parachipteria willmanni*. Mite assemblages in moss on beech stumps and moss on beech trunks (0.5 and 2.0 m) were the most similar and rotting wood and marsh litter differed greatly.

## 1. Introduction

Mites (Acari) are one of the most numerous arachnid groups of species. The number of species distinguished to date is over to 55 thousand [1,2,3], although it is assumed that most mite species have not been discovered yet. Thanks to recent advances in microscopic and molecular techniques, many as several hundred species new to science are being described every year. In turn, species diversity is closely correlated with a specific habitat or microhabitat. In the polar regions, the number of mite species is limited, whereas in the forest environment, a huge diversity is typically observed [4]. Mites may be found practically everywhere, such as in the forest litter and in the soil, on tree stems and foliage of tree crowns, in feeding galleries of insects, in rotting wood, and in ant hills, while some mites are even parasites of mammals and birds. Such habitat selection is a result of many factors, among which a significant role is played by the feeding type, since mites may be parasites, predators, herbivores, or saprophages (detritivores) feeding on decomposing, dead organic matter [4,5]. In view of the specific character of this invertebrate group, mites are increasingly often used as bioindicators of environmental changes, also including forest habitats, e.g., [6,7,8,9,10,11,12,13,14].

Forests in Europe vary greatly as a consequence of tree species diversity, resulting among other things from climate and soil conditions in individual geographic regions and altitude. The European beech (*Fagus sylvatica* L.) is a tree species widely distributed in Europe. The natural range of this species extends in the east from Sweden, Poland, Ukraine, Romania to Bulgaria, through central Europe to France, southern England, and the Iberian Peninsula, largely as a result of climate change following the period of glaciation [15]. A significant impact on the spread of beech seems to have been played by human settlements and landscape transformations, as indicated, e.g., by studies in environmental history [16]. Intensive timber harvesting in beech forests has resulted in a drastic decline in the area covered by this species in Europe. The present growth and preservation of beech stands depend to a considerable extent on forest management, with stand management practices and preservation methods potentially affecting the maintenance of biodiversity [17]. For example, maintenance of an even-aged, single-story stand structure, from which old and rotting trees are removed has a negative effect on mite diversity, among other things [18,19].

One of the primary aims of the European Green Deal [20] is to preserve biodiversity. For this purpose, such documents as the EU Biodiversity Strategy for 2030 [21] and the New EU Forest Strategy for 2030 [22] were developed, imposing new obligations on forestry. In this context, an important role is played by studies concerning biodiversity in the forest environment and the identification of factors determining it.

The eastern limit of the natural range of beech extends through Poland and the experimental site was selected in its vicinity. This site comprised a stand excluded from commercial forest management; thus, it is more natural in character. The aim of this study was to define the effect of microhabitat conditions found in beech forests on the diversity of mite assemblages and their species richness. This knowledge will facilitate the development of more effective methods to preserve the diversity of mites, being bioindicators used during forest management operations. Moreover, in the future, the obtained research results may provide comparative data for acarological studies in beech stands in other regions of Europe.

## 2. Materials and Methods

### 2.1. Field Studies

The study was conducted in the Wronie Forest Reserve (53°18′28″ N, 18°54′09″ E, 115 m a.s.l.), which is the site of a natural Lowland Beech Forest. In this forest, the beech crowns cover 90% of the total area with lower coverage of oak (*Quercus robur* L. and *Q*. *sessilis* L.), ash (*Fraxinus excelsior* L.), maple (*Acer platanoides* L.), Scots pine (*Pinus sylvestris* L.), and larch (*Larix decidua* Mill.). The bush layer is poorly developed and includes elderberry (*Sambucus nigra* L. and *S*. *racemosa* L.), alder backthorn (*Frangula alnus* Mill.) and clusters of sycamore maple (*Acer pseudoplatanus* L.). The forest floor vegetation is weakly developed and includes melic grass (*Melica uniflora* Retz), oak fern (*Phegopteris dryopteris* L. Fée), shield fern (*Thelypteris palustris* Schott), anemone (*Anemone nemorosa* L. and *A*. *ranunculoides* L.), and green pea (*Lathyrus vernus* L.), all of which are typical for beech forest. In the summer, groose grass [*Galium odoratum* (L.) Scop.], millet grass (*Milium effusum* L.), meadowgrass (*Poa nemoralis* L.), and convallaria [*Maianthemum bifolium* (L.) F. W. Schmidt] are present. Depressions are filled with stagnant water over which *Bidentea tripartiti*, *Phragmitetea* and *Alnetea glutinosae* with rush (*Juncus effuses* L.) and reed grass [*Calamagrostis canescens* (Weber) Roth] were grown. The material was collected in May 2011 from various microhabitats such as beech litter, moss on beech litter, moss on beech stumps, rotting beech wood, marsh litter, and moss on beech trunks collected from either 0.5 m or 2 m above the beech litter. Six replicates, each 100 cm^2^ × 5 cm in depth, were taken, except for those from the tree trunks, where the moss was thinner and which were 200 cm^2^ × 2.5 cm deep.

### 2.2. Laboratory Procedures

The collected samples were placed into Tullgren funnels for 14 days (due to the large sample size) and preserved in 96% ethanol. Using a stereoscopic microscope, the extracted mites were classified into systematic groups, i.e., Mesostigmata and Oribatida. In order to identify Mesostigmata (Acari: Parasitiformes), both semi-permanent (using lactic acid) and permanent microslides (using Hoyer’s medium) were prepared. The Oribatida (Acari: Acariformes) were identified at high magnifications (l00–1000×) under a light microscope, preferably with a phase contrast and a differential interference contrast. Prior to the examination, the cuticles were rendered transparent and in freshly collected individuals the internal tissue was removed using concentrated lactic acid (60% lactic acid). The clearing process was performed at room temperature over a course of several days, and sometimes weeks. Oribatid mites were determined at a species level by identifying their key features and original species descriptions, e.g., [23,24]. All the mesostigmatic mites were examined under a light microscope (Zeiss Axioskop 2, Oberkochen, Germany) based on the taxonomic literature, e.g., [25,26]. All the investigated material is deposited at the Department of Biology and Animal Environment, the Bydgoszcz University of Science and Technology, Poland.

### 2.3. Statistical Analyses

Analysis of the results was performed using statistical inference methods [27,28]. The basic statistical descriptors included mean values $\bar{x}$ and standard deviation (±SD). Normality of distribution was tested with the use of the W Shapiro–Wilk test, while equality of variance in different samples was tested with Levene’s test. As the assumptions of variance analysis were not met, nonparametric tests were employed. The Kruskal–Wallis test was applied to inspect the raw data and, in the case of significant differences between means, a multiple comparison test on ranks was employed. Similarities of the communities of the Oribatida and Mesostigmata between microhabitats were analyzed using the unweighted pair group method with arithmetic mean (UPGMA) with Bray–Curtis (percent similarity) coefficients using the full data of studied communities. Correspondence analysis (CA) was used to find the main gradients of microhabitat preferences for selected Oribatida and Mesostigmata species [29,30]. Mite communities were characterized by the Shannon H′ index. The dominance (*D* in %) and constancy (*C* in %) ranges established by Seniczak [31] were used to draw up the dominance structure of the mites. The level of significance for all statistical tests was accepted at α = 0.05. The statistical calculations mentioned above were carried out using the MS Excel 2019 software (Microsoft, Redmond, WA, USA, 2019), Statistica 13.3 (Dell, Round Rock, TX, USA, 2023), PAST 3.2 (Hammer UiO, 2018) and MVSP 3.2 (Multi Variate Statistical Package, Kovach Computing Services 2019) software.

## 3. Results

In collected samples, a total of 144 species were found (78 species of Oribatida, 66 species of Mesostigmata), represented by 74,433 mite individuals (71,124 Oribatida and 3309 Mesostigmata). All the analyzed microhabitats differed in terms of reported mite assemblages. In beech litter, a total of 68 mite species were identified (48 Oribatida and 20 Mesostigmata). No superdominants or eudominants were found, while two species of Oribatid mites were dominants (*Chamobates subglobulus*, *Plathynothrus peltifer*). Among Mesostigmata in this microhabitat, there were superdominants (*Zercon peltatus*) and eudominants (*Zercon gurensis*).

In moss on beech litter, a total of 62 mite species were recorded (48 Oribatida and 14 Mesostigmata). No superdominants were found, whereas single species were reported for the group of eudominants (*Achipteria coleoptrata*) and dominants (*Oppiella nova*). On the other hand, in the Mesostigmata communities, a single species represented the classes superdominants (*Veigaia nemorensis*), eudominants (*Paragamasus lapponicus*) and dominants (*Zercon gurensis*).

In moss on beech stumps, 72 mite species were detected (53 Oribatida and 19 Mesostigmata). *Parachipteria willmanni* was a superdominant in this microhabitat. Similarly, among Mesostigmata, only *Zercon peltatus* was superdominant in this microhabitat.

A different assemblage structure was recorded in rotting wood, where 65 mite species were identified (34 Oribatida and 31 Mesostigmata). One species was found in the group of eudominants (*Oppiella uliginosa*) as well as two dominant species (*Chamobates cuspidatus*, *Quadroppia quadricarinata*). In the Mesostigmata communities, only one dominant species (*Zercon peltatus*) was found.

In marsh litter, a total of 29 mite species were reported (15 Oribatida and 14 Mesostigmata), among which one was an eudominant (*Hydrozetes thienemanni*) and two species were dominants (*Pilogalumna tenuiclava*, *Zetomimus furcatus*). Among Mesostigmata, superdominant (*Platyseius italicus*), eudominant (*Cheiroseius* sp.) and dominant (*Platyseius subglaber*) occurred in this microhabitat.

Moreover, analyses covered mite assemblages found in moss growing on beech trunks at two heights.

At 0.5 m above the litter, 56 mite species were recorded (40 Oribatida and 16 Mesostigmata). In this mite group, *Parachipteria willmanni* was a superdominant and it was also abundant in moss on beech stumps, whereas dominants included *Oribatula exilis* and *Neobrachychthonius magnus*. The superdominant was also *Zercon peltatus* (Mesostigmata) and the dominants *Veigaia nemorensis* and *Celaenopsis badius* (Mesostigmata).

In samples collected at a height of 2.0 m above the litter, similar to those collected at a lower height, *Parachipteria willmanni* was superdominant, while *Oribatula exilis* was eudominant. Additionally, analysis of the numbers of species classified as subrecedents, i.e., found incidentally, showed that their number is highest in moss on stumps (58 species), in moss growing on beech trunks at a height of 0.5 m a.l. (57) and in beech litter (54), whereas it was lowest in marsh litter (18). In Mesostigmata communities at 2.0 m above the litter superdominant was *Zercon peltatus* and the dominant *Veigaia nemorensis*. Among Mesostigmata, subrecedents showed that their number is highest in beech litter (17) and lowest in marsh litter (5) (Supplementary Materials Tables S1–S3).

In view of the considerable number of species classified as subrecedents, i.e., those found in a specific microhabitat incidentally, statistical analyses included only the mite species with a major share in the collected material (mean density < 20). This facilitated a more precise identification of the character of mite assemblages depending on a given microhabitat (Table 1).

**Figure 1:**
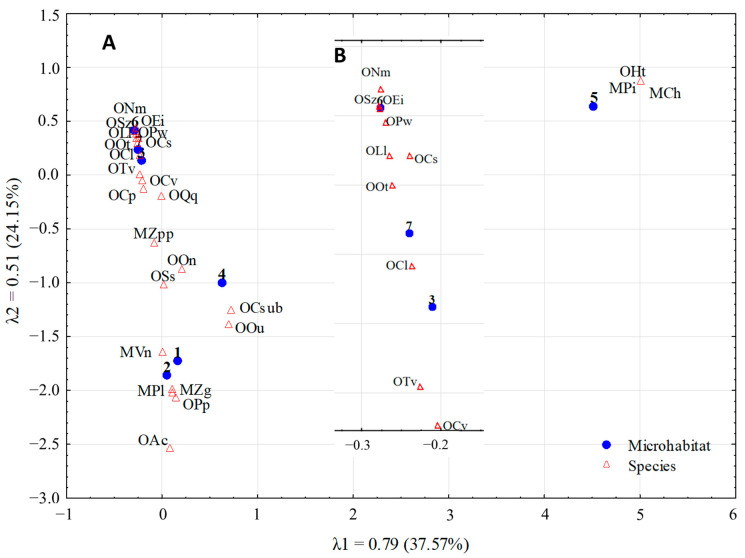
(**A**) Biplot of the first 2 axes of a correspondence analysis (CA) of 25 species and 7 microhabitats of beech forest in the Wronie Reserve. (**B**) Enlarged top part of (**A**). Microhabitats: 1—Beach litter, 2—Moss on beach litter, 3—Moss on beach stumps, 4—Rotting beach wood, 5—Marsh litter, 6—Moss on beach trunks (0.5 m a.l.), 7—Moss on beach trunks (2.0 m a.l.). Species: OAc—*Achipteria coleoptrata*, MCh—*Cheiroseius* sp., OCl—*Carabodes labyrinthicus*, OCp—*Chamobates pusillus*, OCs—*Chamobates spinosus*, OCsub—*Chamobates subglobulus*, OCv—*Chamobates voigtsi*, OEi—*Euremaeus intermedius*, OHt—*Hydrozetes thienemanni*, OLl—*Licneremaeus licnophorus*, ONm—*Neobrachychthonius magnus*, OOt—*Oribatula tibialis*, OOn—*Oppiella nova*, OOu—*Oppiella uliginosa*, OQq—*Quadroppia quadricarinata*, OPw—*Parachipteria willmanni*, MPl—*Paragamasus lapponicus*, MPi—*Platyseius italicus*, OPp—*Plathynothrus peltifer*, OSs—*Suctobelbella subcornigera*, OSz—*Sellnickochthonius zelewaensis*, OTv—*Tectocepheus velatus*, MVn—*Veigaia nemorensis*, MZg—*Zercon gurensis*, MZpp—*Zercon peltatus peltatus*.

Eigenvalues for both axes (CA analysis, Figure 1) are statistically significant [32] and for the first and second dimensions, they are λ1 = 0.79 and λ2 = 0.51, respectively. The eigenvalues of the axes indicate that the gradient represented by both the first and second ordinate axis differentiates the occurrence of species. The two dimensions explain 61.72% of the overall variability in the occurrence of Oribatida and Mesostigmata species. The marsh litter microhabitat contributed most to the definition of the two-variate space.

Among the investigated microhabitats, we may distinguish groups of similar density structures for selected species of Oribatida and Mesostigmata. The first group consists of microhabitats of moss on beech stumps, moss on beech trunks (0.5 m a.l.), and moss on beech trunks (2.0 m a.l.). The second group comprises the microhabitat of rotting beech wood, the third includes microhabitats of beech litter and moss on beech forest soil, and the fourth includes marsh litter.

The distances between points representing the density of individual Oribatida and Mesostigmata species in the two-factor space are similar. The analyzed species—*Tectocepheus velatus* (OTv), *Chamobates voigtsi* (OCv), *Chamobates pusillus* (OCp) *Quadroppia quadricarinata* (OQq) as well as *Hydrozetes thienemanni* (OHt), *Platyseius italicus* (MPi), *Cheiroseius* sp. (MCh) and *Achipteria coleoptrata* (Oac)—vary in the way they diversify investigated microhabitats. Based on this analysis, it may be stated that the microhabitats of moss on beech stumps, moss on beech trunks (0.5 m a.l.) and moss on beech trunks (2.0 m a.l.) are significantly distinguished from the other studied habitats in view of the density of *Sellnickochthonius zelewaensis* (OSz), *Neobrachychthonius magnus* (ONm), *Euremaeus intermedius* (OEi), *Parachipteria willmanni* (OPw), *Licneremaeus licnophorus* (OLl), *Oribatula tibialis* (OOt), *Chamobates spinosus* (OCs), *Carabodes labyrinthicus* (OCl), *Tectocepheus velatus* (OTv), and *Chamobates voigtsi* (OCv). These microhabitats are distinct in terms of the different density structures of individual oribatid mite species from the other microhabitats. Moreover, correspondence analysis (CA) showed that the two dimensions distinctly differentiate fauna of Oribatida and Mesostigmata from moss on beech stumps, moss on beech trunks (0.5 m a.l.) and moss on beech trunks (2.0 m a.l.) from the fauna of beech litter, moss on beech soil as well as rotting beech wood and marsh litter.

Species such as *Hydrozetes thienemanni* (OHt), *Platyseius italicus* (MPi), and *Cheiroseius* sp. (MCh) show marked preference in relation to the microhabitat of marsh litter. Similarly, *Sellnickochthonius zelewaensis* (OSz), *Neobrachychthonius magnus* (ONm), *Euremaeus intermedius* (OEi), *Parachipteria willmanni* (OPw), *Licneremaeus licnophorus* (OLl), *Oribatula tibialis* (OOt), *Chamobates spinosus* (OCs), and *Carabodes labyrinthicus* (OCl) show a definite preference in relation to moss on beech trunks (0.5 m a.l.), moss on beech trunks (2.0 m a.l.) and moss on beech stumps. Marked preferences to marsh litter and moss on beech soil were observed for *Veigaia nemorensis* (MVn), *Paragamasus lapponicus* (MPl), *Zercon gurensis* (MZg), *Plathynothrus peltifer* (OPp), and *Achipteria coleoptrata* (OAc). In turn, *Zercon peltatus peltatus* (MZpp), *Oppiella nova* (OOn), and *Suctobelbella subcornigera* (OSs) show shared preferences in relation to most habitats. *Chamobates subglobulus* (OCsub) and *Oppiella uliginosa* (OOu) also exhibit shared preferences related to the studied habitats, with rotting beech wood being most preferable (Figure 1).

**Figure 2:**
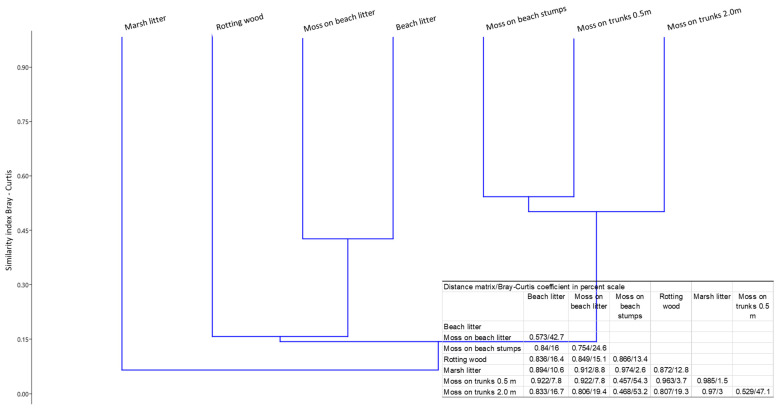
Similarities (Bray–Curtis coefficient in percent scale) of the Oribatida and Mesostigmata communities between microhabitats of beech forest in the Wronie Reserve.

Similarities and differences between the mite assemblages in selected microhabitats of beech stands may also be assessed based on cluster analysis (Figure 2). Recorded results are consistent with those given above. High similarity was found between the mite assemblages colonizing moss of trunks (0.5 m a.l. and 2.0 m a.l.) and moss on beech stumps. Another group comprised assemblages colonizing beech litter and moss on beech litter. In turn, assemblages found in rotting wood and marsh litter were distinctly different.

## 4. Discussion

Mite assemblages found in the forest environment were discussed in numerous publications, which analyzed their microhabitat selection, e.g., [19,26,33,34,35]. However, mite assemblages colonizing diverse microhabitats in beech stands have not been extensively investigated. Migge et al. [36] studied the density of oribatid mites in the litter and soil of beech and spruce stands in northern Germany, reporting on average 160,000 individuals per 1 m^2^. Those authors recorded 68 mite species and stated that some of them, e.g., *Hypochthonius rufulus* and *Nanhermannia coronata*, were found more frequently in beech rather than spruce stands. These results may not be reliably referred to those reported in this study, since although *Hypochthonius rufulus* was also reported here, it was not numerous and it was classified to the group of recedents (moss on beech litter) or subrecedents (beech litter).

In Poland, studies on mites in beech stands have been conducted, e.g., in the Karkonosze National Park and in *Luzulo-Fagetum* plant communities [37]. *Veigaia nemorensis* and *Gamasellus montanus* (species common in the southern mountainous region of Poland) were dominant species in beech litter. In that national park, an experiment was also carried out, consisting of spreading beech leaves under the canopy of a spruce stand. It was one of the stages in the transformation of spruce stands into beech stands. In beech leaves, such species as *Pachylaelaps bellicossus*, *Geholaspis pauperior* and *Pachylaelaps furcifer* were dominant [38]. Thus, it may be stated that compared to this paper completely different mite assemblages were identified in that study.

Maraun et al. [39] investigated the effects of mechanical perturbations on soil microarthropod communities (oribatid mites and collembolans) in a modern beech forest on sandstone. Those authors disturbed the soil matrix by sieving and mixing the litter and soil of the modern profile. The densities of most groups of oribatid mites declined in the disturbance treatments. Desmonomata (*Nothrus silvestris*, *Platynothrus peltifer*, *Nanhermannia nana*, *N*. *coronata*) were the only group of oribatid mites that benefited from intermediate disturbance but not from the strongest one. In contrast, in this study, only *N*. *silvestris* was detected, but it was found incidentally (in the group of recedents or subrecedents).

Other research results concerned mites from the family Ascidae in beech stands, as presented by Gwiazdowicz [26]. The author showed 23 species, among which the most numerous included *Cheiroseius necorniger*, *Lasioseius ometes*, *Lasioseius muricatus*, *Zerconopsis remiger,* and *Cheiroseius borealis*. Only *Z*. *remiger* was recorded in microhabitats analyzed in this study; however, it was an incidental species, as only four individuals were found. In turn, Sabbatini Peverieri et al. [40] described assemblages of mesostigmatic mites in beech stands in central Italy, reporting 65 species. However, the structure of mite assemblages was completely different from that presented in this study. While the presence of some species such as *Veigaia nemorensis* and *Pergamasus lunulata* was confirmed, they are relatively common species, found in many microhabitats, and in the analyzed study, they were incidental.

Kamczyc et al. [41] analyzed the biomass of mesostigmatic mites in decayed wood (coarse woody debris, CWD) in beech stands in Poland. Those authors stated that decaying logs were dominated by *Janietella pulchella*, whereas in the soil, it was *Veigaia nemorensis*. In this study, only *Zercon peltatus* was reported in rotting wood microhabitat, which was dominant. *Veigaia nemorensis* was superdominant in moss on beach litter and in samples collected at a height of 0.5 and 2.0 m above the litter it was dominant.

It may be difficult to definitely state the exact reasons for such a marked diversity of mite assemblages in beech stands. This question might be answered by studies comparing microhabitat conditions, e.g., species of fungi or nematodes, constituting the feed base for mites.

In the analyzed material, *Parachipteria willmanni* was the most numerous species; it was classified as a superdominant in moss on beech stumps and moss on beech trunks (0.5 and 2.0 m a.l.). *Parachipteria willmanni* is considered a Holarctic species and prefers rather wet habitats. It was collected in damp to wet moor humus, damp forests near birch, and alder marshes and flooded meadows [3,42,43,44]. The group of eudominants included *Achipteria coleoptrata* (in moss on beech litter), *Oppiella uliginosa* (in rotting wood), *Hydrozetes thienemanni* (in marsh litter) and *Oribatula exilis* (moss on beech trunks at 2.0 m a.l.). *Achipteria coleoptrata* is a Holarctic species. It prefers moderately moist environments, fresh and moist meadows and forests, and medium to high humus content in the soil [43,44,45]. *Oppiella uliginosa* is found in Central Europe and prefers moss cushions in beech forests. The ecology of this species is not well understood [44]. *Hydrozetes thienemanni* inhabits Holarctic or Boreal regions [3,44] and it is a hygrophilous species. It is found in lakes, in ponds, and in standing and slowly flowing water, mainly on submergent vegetation, but also on detritus [24,46]. It is also shown from waterlogged organic matter and proper soil of reedy thickets, alder wood, eutrophic fens, and flooded meadows, as well as Carex bogs [43,47,48,49]. In turn, *Oribatula exilis* is a Holarctic species, shown throughout Europe, Asia and North America [43,44]. It inhabits, in large numbers, various microhabitats in beech forests [50]. It is often found in mosses and on tree bark, including in urban environments [44,51,52]. It has also been observed in xerothermic pine forests [53], fields, meadows and in the shoreline vegetation of water bodies [54].

## 5. Conclusions

Summing up, it may be stated that the species structure of mite communities as well as the population size of mites differed considerably depending on the analyzed microhabitat. It is likely to be modified by the different conditions, e.g., humidity and/or temperature, which determine the food base for mites. Reported findings differed considerably from results given by other authors for other geographical regions. An answer to the question concerning the genesis of these differences may be provided only by further research on the main factors (both abiotic and biotic), influencing the species structure of mite communities in beech stands. This is particularly important in view of climate warming and changes in the geographical range of beech, with mites potentially being good bioindicators, facilitating a more accurate analysis in relation to directions of environmental changes.

## Figures and Tables

**Table 1:** Density ^1^ of selected species of Oribatida (O) and Mesostigmata (M), dominance (*D*, %), constancy (*C*, %), indices in certain microhabitats of beech forest in the Wronie Reserve. Ad—adult, Juv—juveniles, Tot—totally. Species with mean density < 20 are listed below. For other species of studied mites, see supplemetary data.

Species		Beech Litter	Moss on Beech Litter	Moss on Beech Stumps	Rotting Wood	Marsh Litter	Moss on Beech Trunks		Mean	ANOVA Rang Kruskal–Wallis	
							0.5 m a.l. ^3^	2.0 m a.l. ^3^		*H*	*p*
*Parachipteria willmanni* van der Hammen, 1952 (O) Achipteriidae Thor, 1929	Ad	0	0	562.8	16.0	0	508.5	137.0	174.9		
	Juv	0	0	689.8	16.2	0	1653.3	650.0	429.9		
	Tot	0 a ^2^	0 a	1252.6 ± 780.0 b	32.2 ± 27.0 ab	0 a	2161.8 ± 879.0 b	787.0 ± 623.1 ab	604.8	38.66	0.000
	*D*	0	0	40.5	6.5	0	46.0	41.4	-		
	*C*	0	0	100	100	0	100	100	-		
*Oribatula exilis* (Nicolet, 1855) (O) Oribatulidae Thor, 1929	Ad	5.5	3.5	60.3	1.7	0.5	758.5	483.2	187.6		
	Juv	32.8	42.2	36.7	0.3	0	135.8	54.3	43.1		
	Tot	38.3 ± 26.5 ab	45.7 ± 53.5 ab	97.0 ± 83.4 ab	2.0 ± 1.6 a	0.5 ± 0.6 a	894.3 ± 548.7 b	537.5 ± 611.2 b	230.7	32.42	0.000
	*D*	5.2	5.1	3.1	0.4	0.1	19.0	28.3	-		
	*C*	100	100	100	83	50	100	100	-		
*Neobrachychthonius magnus* Moritz, 1976 (O) Brachychthoniidae Thor, 1934	Ad	0	0.2	106	0	0.8	111.6	0	31.2		
	Juv	0	0.8	133.2	0	0	418.7	0	78.9		
	Tot	0 a	1.0 ± 2.4 ab	239.2 ± 275.6 ab	0 a	0.8 ± 2.0 ab	530.3 ± 421.0 b	0 a	110.2	36.33	0.000
	*D*	0	0.1	7.7	0	0.2	11.3	0	-		
	*C*	0	17	100	0	17	100	0	-		
*Oppiella nova* (Oudemans, 1902) (O) Oppiidae Grandjean, 1951	Ad	30.5	148.0	194.5	17.8	25.8	0.2	46.8	66.1		
	Juv	0	0	0.1	0	0	0	0	<0.1		
	Tot	30.5 ± 36.9 ab	148.0 ± 140.3 a	194.6 ± 207.9 a	17.8 ± 15.1 ab	25.8 ± 56.0 ab	0.2 ± 0.4 b	46.8 ± 43.4 ab	66.2	25.50	0.001
	*D*	4.2	16.6	6.3	3.6	5.6	<0.1	2.5	-		
	*C*	100	100	100	83	83	17	100	-		
*Chamobates pusillus* (Berlese, 1895) (O) Chamobatidae Grandjean, 1954	Ad	7.9	4.7	7.7	0	0	6.8	11.1	5.5		
	Juv	16.3	12.5	155.3	0	0	9.0	73.2	38.0		
	Tot	24.2 ± 16.5 ab	17.2 ± 16.5 ab	163.0 ± 115.6 a	0 b	0 b	15.8 ± 38.8 bc	84.3 ± 82.0 ac	43.5	31.46	0.000
	*D*	3.3	1.9	5.3	0	0	0.3	4.4	-		
	*C*	100	100	100	0	0	17	100	-		
*Quadroppia quadricarinata* (Michael, 1885) (O) * Quadroppiidae Balogh, 1983	Tot	0.3 ± 0.8 ac	1.0 ± 1.7 abc	141.7 ± 106.1 b	71.5 ± 39.8 ab	0 c	0.2 ± 0.4 ac	73.7 ± 53.1 ab	41.2	34.53	0.000
	*D*	0.1	0.1	4.6	14.4	0	<0.1	3.9	-		
	*C*	17	33	100	100	0	17	100	-		
*Chamobates spinosus* Sellnick, 1928 (O) Chamobatidae Grandjean, 1954	Ad	0	0	67.7	9.7	0	76.3	0	22.0		
	Juv	0	0	38.5	0.6	0	87.0	0	18.0		
	Tot	0 a	0 a	106.2 ± 151.2 ab	10.3 ± 7.3 ab	0 a	163.3 ± 272.8 b	0 a	40.0	34.26	0.000
	*D*	0	0	3.4	2.1	0	3.5	0	-		
	*C*	0	0	100	100	0	83	0	-		
*Chamobates subglobulus* (Oudemans, 1900) (O) Chamobatidae Grandjean, 1954	Ad	3.0	1.4	4.8	5.3	19.1	13.0	4.2	7.2		
	Juv	129.3	29.8	12.2	5.7	16.7	30.3	3.5	32.5		
	Tot	132.3 ± 71.3 a	31.2 ± 30.8 ab	17.0 ± 8.3 ab	11.0 ± 7.2 b	35.8 ± 48.5 ab	43.3 ± 14.5 ab	7.7 ± 5.0 b	39.7	23.04	0.001
	*D*	18.0	3.5	0.6	2.2	7.7	0.9	0.4	-		
	*C*	100	100	100	100	83	100	100	-		
*Suctobelbella subcornigera* (Forsslund, 1941) (O) Suctobelbidae Jacot, 1938	Ad	44.8	53.3	66.0	34.5	0	0	60.7	37.1		
	Juv	3.2	0	0	0	0	0	0	0.4		
	Tot	48.0 ± 39.2 ab	53.3 ± 18.0 a	66.0 ± 48.0 a	34.5 ab	0 b	0 b	60.7 ± 58.8 ab	37.5	24.06	0.001
	*D*	6.5	6.0	2.1	7.0	0	0	3.2	-		
	*C*	100	100	100	100	0	0	67	-		
*Tectocepheus velatus* (Michael, 1880) (O) Tectocepheidae Oudemans, 1900	Ad	2.5	57.2	123.7	4.0	0	297.2	22.0	72.3		
	Juv	0.5	23.3	35.5	0.5	0	35.8	9.7	15.1		
	Tot	3.0 ± 2.9 a	80.5 ± 39.5 b	159.2 ± 140.1 b	4.5 ± 4.4 ac	0 ac	333.0 ± 377.1 bc	31.7 ± 40.1 ab	87.4	31.28	0.000
	*D*	0.4	9.0	5.1	0.9	0	7.1	1.7	-		
	*C*	67	100	100	83	0	100	83	-		
*Achipteria coleoptrata* (Linne, 1758) (O) Achipteriidae Thor, 1929	Ad	13.2	132.1	0	0	0	0	0	20.8		
	Juv	34.8	67.7	0	0	0	0	0	14.6		
	Tot	48.0 ± 22.4 a	199.8 ± 263.4 a	0 b	0 b	0 b	0 b	0 b	35.4	39.66	0.000
	*D*	6.5	22.4	0	0	0	0	0	-		
	*C*	100	100	0	0	0	0	0	-		
*Chamobates voigtsi* (Oudemans, 1902) (O) Chamobatidae Grandjean, 1954	Ad	37.0	5.2	7.0	0.7	0	40.4	0.7	13.0		
	Juv	2.3	0	0	0	0	129.8	0	18.9		
	Tot	39.3 ± 27.5 ac	5.2 ± 7.0 abc	7.0 ± 8.4 abc	0.7 ± 1.6 ab	0 b	170.2 ± 146.6 c	0.7 ± 1.2 ab	31.9	28.22	0.000
	*D*	5.4	0.6	0.2	0.1	0	3.6	<0.1	-		
	*C*	100	50	50	17	0	100	33	-		
*Euremaeus intermedius* Mihelčič,1963 (O) Eremaeidae Oudemans, 1900	Ad	1.2	0.3	0.3	0	0	53.0	24.5	11.6		
	Juv	0.8	0	0	0.5	0	55.5	72.2	18.4		
	Tot	2.0 ± 4.9 ab	0.3 ± 0.8 a	0.3 ± 0.8 a	0.5 ± 0.6 ab	0 a	108.5 ± 151.5 b	96.7 ± 159.9 b	30.0	20.98	0.002
	*D*	0.3	<0.1	<0.1	0.1	0	2.3	5.1	-		
	*C*	17	17	17	50	0	100	100	-		
*Zercon peltatus peltatus* C. L. Koch, 1836 (M) Zerconidae Canestrini, 1891	Ad	36.3	0	16.2	3.7	0 a	12.8	10.7	11.4		
	Juv	30.4	0	57.3	3.7	0	16.0	21.5	18.4		
	Tot	66.7 ± 49.1 a	0 b	73.5 ± 89.8 ab	7.4 ± 7.9 ab	0 b	28.8 ± 18.4 ab	32.2 ± 41.9 ab	29.8	25.34	0.001
	*D*	9.1	0	2.4	1.5	0	0.6	1.7	-		
	*C*	100	0	100	83	0	100	50	-		
*Sellnickochthonius zelawaiensis* (Sellnick, 1928) (O) Brachychthoniidae Thor, 1934	Ad	0	0	68.3	0	0	107.2	0	25.1		
	Juv	0	0	5.5	0	0	7.8	0	1.9		
	Tot	0 a	0 a	73.8 ± 61.3 b	0 a	0 a	115.0 ± 114.5 b	0 a	27.0	27.59	0.000
	*D*	0	0	2.4	0	0	2.5	0	-		
	*C*	0	0	100	0	0	67	0	-		
*Carabodes labyrinthicus* (Michael, 1879) (O) * Carabodidae C.L. Koch, 1843	Tot	0 a	3.5 ± 4.3 ab	127.0 ± 152.5 b	0.5 ± 0.6 ac	0 a	27.5 ± 29.2 bc	14.7 ± 11.7 ab	24.7	34.06	0.000
	*D*	0	0.4	4.1	0.1	0	0.6	0.8	-		
	*C*	0	83	100	50	0	100	100	-		
*Hydrozetes thienemanni* Strenzke, 1943 (O) Hydrozetidae Grandjean, 1954	Ad	0	0	0	0	146.4	0	0	20.9		
	Juv	0	0	0	0	2.6	0	0	0.4		
	Tot	0 a	0 a	0 a	0 a	149.0 ± 169.8 b	0 a	0 a	21.3	40.69	0.000
	*D*	0	0	0	0	32.1	0	0	-		
	*C*	0	0	0	0	100	0	0	-		
*Oppiella uliginosa* (Willmann, 1919) (O) Oppiidae Grandjean, 1951	Ad	0	0	0	117.0	0	0	0	16.7		
	Juv	0	0	0	0.1	0	0	0	<0.1		
	Tot	0 a	0 a	0 a	117.1 ± 69.2 b	0 ab	0 ab	0 ab	16.7	40.69	0.000
	*D*	0	0	0	23.6	0	0	0	-		
	*C*	0	0	0	100	0	0	0	-		
*Plathynothrus peltifer* (C.L. Koch, 1839) (O) Camisiidae Oudemans, 1900	Ad	10.5	7.0	0.5	1.3	0	1.9	1.7	3.3		
	Juv	63.3	15.3	1.3	4.0	0	2.8	4.3	13.0		
	Tot	73.8 ± 61.9 a	22.3 ± 13.5 ab	1.8 ± 2.1 b	5.3 ± 6.1 ab	0 cb	4.7 ± 4.5 ab	6.0 ± 5.9 ab	16.3	29.62	0.000
	*D*	10.0	2.5	0.1	1.1	0	0.1	0.3	-		
	*C*	100	100	50	67	0	83	67	-		
*Licneremaeus licnophorus* (Michael, 1882) (O) * Licneremaeidae Gradjean, 1931	Tot	0 a	0 a	63.0 ± 129.3 b	0 a	0 a	46.7 ± 81.4 b	0 a	15.7	14.41	0.025
	*D*	0	0	2.0	0	0	1.0	0	-		
	*C*	0	0	33	0	0	50	0	-		
*Suctobelbella subtrigona* (Oudemans, 1916) (O) * Suctobelbidae Jacot, 1938	Tot	0 a	8.2 ± 7.3 ab	77.2 ± 63.0 b	0 a	0 a	0 a	17.2 ± 30.2 ab	14.6	24.16	0.001
	*D*	0	0.9	2.5	0	0	0	0.9	-		
	*C*	0	67	83	0	0	0	33	-		
*Chamobates cuspidatus* (Michael, 1884) (O) Chamobatidae Grandjean, 1954	Ad	0	0	0	70.3	25.8	0	0	13.7		
	Juv	0	0	0	3.2	0.4	0	0	0.5		
	Tot	0 a	0 a	0 a	73.5 ± 80.7 b	26.2 ± 24.8 ab	0 a	0 a	14.2	36.07	0.000
	*D*	0	0	0	14.8	5.6	0	0	-		
	*C*	0	0	0	100	83	0	0	-		
*Suctobelba trigona* (Michael, 1888) (O) * Suctobelbidae Jacot, 1938	Tot	0 a	19.0 ± 19.5 b	64.8 ± 116.5 ab	1.5 ± 1.4 ab	0 a	0 a	14.0 ± 24.2 ab	14.1	23.65	0.001
	*D*	0	2.1	2.1	0.3	0	0	0.7	-		
	*C*	0	100	67	67	0	0	50	-		
*Nothrus silvestris* Nicolet, 1855 (O) Nothridae Berlese, 1896	Ad	12.5	10.8	3.5	0	0	1.3	0	4.0		
	Juv	10.0	28.8	12.8	0	0	1.0	0	7.5		
	Tot	22.5 ± 24.6 ab	39.7 ± 29.2 a	16.3 ± 26.1 ab	0 b	0 b	2.3 ± 2.7 ab	0 b	11.5	31.54	0.000
	*D*	3.1	4.4	0.5	0	0	0.1	0	-		
	*C*	83.0	100	100	0	0	50	0	-		
*Pilogalumna tenuiclava* (Berlese, 1908) (O) Galumnidae Jacot,1925	Ad	0	0	0	0	3.0	0	0	0.4		
	Juv	0	0	0	0	72.0	0	0	10.3		
	Tot	0 a	0 a	0 a	0 a	75.0 ± 171.7 b	0 a	0 a	10.7	18.89	0.005
	*D*	0	0	0	0	16.2	0	0	-		
	*C*	0	0	0	0	50	0	0	-		
*Veigaia nemorensis* (C. L. Koch, 1839) (M) Veigaiaidae Oudemans, 1939	Ad	8.3	19.7	4.2	0.7	0	4.3	1.7	5.6		
	Juv	3.9	15.0	5.8	1.8	0	1.2	3.7	4.5		
	Tot	12.2 ± 9.9 ab	34.7 ± 18.5 a	10.0 ± 7.8 ac	2.5 ± 3.8 bc	0 bc	5.5 ± 3.0 ac	5.4 ± 7.5 ac	10.0	25.53	0.001
	*D*	1.7	3.9	0.3	0.5	0	0.1	0.3	-		
	*C*	83	100	100	67	0	100	50	-		
*Carabodes femoralis* (Nicolet, 1855) (O) * Carabodidae C.L. Koch, 1843	Tot	0.7 ± 1.0 ab	3.5 ± 3.6 ab	29.8 ± 23.8 a	13.8 ± 5.5 a	0 b	0.8 ± 1.6 ab	15.2 ± 16.0 ab	9.1	22.82	0.001
	*D*	0.1	0.4	1.0	2.8	0	<0.1	0.8	-		
	*C*	33	67	83	100	0	33	67	-		
*Zetomimus furcatus* (Pearce & Warburton, 1906) (O) Zetomimidae Shaldybina, 1966	Ad	0	0	0	4.8	0.2	0	0	0.7		
	Juv	0	0	0	0	53.5	0	0	7.6		
	Tot	0 a	0 a	0 a	4.8 ± 1.7 ab	53.7 ± 63.3 b	0 a	0 a	8.3	35.37	0.000
	*D*	0	0	0	1.0	11.6	0	0	-		
	*C*	0	0	0	100	83	0	0	-		
*Zercon gurensis* Mihelčič, 1962 (M) Zerconidae Canestrini, 1891	Ad	24.8	2.5	0.2	0.3	0	1.7	0.2	4.2		
	Juv	8.0	6.8	3.8	0.2	0	1.2	0	2.9		
	Tot	32.8 ± 17.5 a	9.3 ± 6.4 ab	4.0 ± 6.8 abc	0.5 ± 0.8 bc	0 c	2.9 ± 3.4 ac	0.2 ± 0.4 bc	7.1	27.20	0.000
	*D*	4.5	1.0	0.1	0.1	0	0.1	<0.1	-		
	*C*	100	100	50	33	0	67	17	-		
*Pergalumna nervosa* (Berlese, 1914) (O) Galumnidae Jacot, 1925	Ad	1.0	2.3	6.0	0.5	0	0	0	1.4		
	Juv	19.0	17.4	0.3	0	0	0	0	5.2		
	Tot	20.0 ± 14.5 a	19.7 ± 14.3 a	6.3 ± 13.1 ab	0.5 ± 0.8 ab	0 b	0 b	0 b	6.6	32.91	0.000
	*D*	2.7	2.2	0.2	0.1	0	0	0	-		
	*C*	100	100	67	33	0	0	0	-		
*Eremaeus hepaticus* C.L. Koch, 1835 (O) Eremaeidae Oudemans, 1900	Ad	3.3	0.8	0	0	0	3.3	0	1.1		
	Juv	29.2	2.7	0.5	0	0	1.7	0.5	4.9		
	Tot	32.5 ± 40.1 a	3.5 ± 7.6 ab	0.5 ± 1.2 ab	0 b	0 b	5.0 ± 5.1 ab	0.5 ± 1.2 ab	6.0	27.08	0.000
	*D*	4.4	0.4	<0.1	0	0	0.1	<0.1	-		
	*C*	100	33	17	0	0	83	17	-		
*Platyseius italicus* (Berlese, 1905) (M) Blattisociidae Garman, 1948	Ad	0	0	0	0	28.3	0	0	4.0		
	Juv	0	0	0	0	13.7	0	0	2.0		
	Tot	0 a	0 a	0 a	0 a	42.0 ± 37.5 b	0 ab	0 ab	6.0	33.07	0.000
	*D*	0	0	0	0	9.1	0	0	-		
	*C*	0	0	0	0	83	0	0	-		
*Nanhermania nana* (Nicolet, 1855) (O) Nanhermanniidae Sellnick, 1928	Ad	0.3	9.8	1.2	0	0	0	3.7	2.1		
	Juv	0.5	11.2	3.8	0	0	0	3.3	2.7		
	Tot	0.8 ± 1.2 ab	21.0 ± 22.1 a	5.0 ± 6.8 ab	0 b	0 b	0 b	7.0 ± 8.7 a	4.8	25.74	0.001
	*D*	0.1	2.4	0.2	0	0	0	0.4	-		
	*C*	50	100	67	0	0	0	67	-		
*Hypochthonius rufulus* C.L. Koch, 1835 (O) Hypochthoniidae Berlese, 1910	Ad	0.5	12.5	0	0	0	0	0	1.9		
	Juv	1.8	17.5	0	0	0	0	0	2.8		
	Tot	2.3 ± 5.7 a	30.0 ± 22.4 a	0 b	0 b	0 b	0 b	0 b	4.7	35.32	0.000
	*D*	0.3	3.4	0	0	0	0	0	-		
	*C*	17	100	0	0	0	0	0	-		
*Cheiroseius* sp. (M) Blattisociidae Garman, 1948	Ad	0	0	0	0	17.2	0	0	2.5		
	Juv	0	0	0	0	4.0	0	0	0.6		
	Tot	0 a	0 a	0 a	0 a	21.2 ± 16.9 b	0 ab	0 ab	3.0	40.70	0.000
	*D*	0	0	0	0	4.6	0	0	-		
	*C*	0	0	0	0	100	0	0	-		

* species without juveniles stages. ^1^—Mean density [individuals in 500 cm^3^ ± SD (standard deviation)]; ^2^—the same letter means insignificant difference between microhabitats at *p* ≤ 0.05; ^3^—a.l.—above the litter.

## Data Availability

The raw data supporting the conclusions of this article will be made available by the authors on request.

## Supplementary Materials

The following supporting information can be downloaded at: https://www.mdpi.com/article/10.3390/insects16040364/s1, Table S1: Density 1 of communities of Oribatida (O) and Mesostigmata (M), species numbers (S) and Shannon index (H’) in certain microhabitats of beech forest in the Wronie Reserve.; Table S2: Density 1 of selected species of Oribatida (O) and Mesostigmata (M), dominance (D, %), constancy (C, %), indices in certain microhabitats of beech forest in the Wronie Reserve. Species with mean density < 20 are included. Table S3: Domination structure of Mesostigmata communities in chosen microhabitats of beech forest in Wronie Reserve. Domination index of species is given in brackets.
