# Supplementary material for: Microhabitat Selectivity of Mites (Acari) in a Natural Lowland Beech Forest (Melico-Fagetum) in Wronie Reserve (Poland)
Source: Insects. 2025 Apr 1;16(4):364. doi: 10.3390/insects16040364 (PMC12028256; doi:10.3390/insects16040364)
Supplement: Supplementary file 1 [file insects-16-00364-s001.zip › insects-3530621-supplementary.pdf]

Table S1. Density [1] of communities of Oribatida (O) and Mesostigmata (M), species numbers (*S*) and Shannon index (*H'*) in certain microhabitats of beech forest in the Wronie Reserve.

Mites and characters		Beech litter	Moss on beech litter	Moss on beech stumps	Rotting wood	Marsh litter	Moss on beech trunks	
							0.5 m a. l.[2]	2.0 m a. l.[2]
Oribatida	*S*	48	48	53	34	15	40	37
	range	20-32	25-34	29-37	20-26	1-13	18-27	9-31
Shannon index	*H'*	2.73 ± 0.07	2.66 ± 0.07	2.30 ± 0.06	2.48 ± 0.06	1.68 ± 0.07	1.75 ± 0.07	1.80 ± 0.07
Mesostigmata	*S*	20	14	19	31	14	16	22
	range	3-14	5-8	7-12	0-18	4-7	4-9	0-17
Shannon index	*H'*	1.69 ±0.06	1.45 ±0.06	1.33 ±0.04	2.94 ±0.06	1.43 ±0.06	1.52 ±0.05	1.54 ±0.05
Total	*S*	68	62	72	65	29	56	59
	range	23-43	31-41	37-45	25-41	8-18	23-36	10-48
Shannon index	*H'*	3.02	2.84	2.44	2.81	2.13	1.79	1.92
Total species number				144 (78(O), 66(M))				
Common species				4(O), 0(M)				
Exclusive species		4(O), 8(M)	1(O), 1(M)	3(O), 2(M)	3(O), 14(M)	5(O), 7(M)	1(O), 2(M)	0(O), 4(M)

[1] – Mean density [individuals in 500 cm[3] ± SD (standard deviation)]; [2] – a. l. – above the litter

Table S2. Density [1] of selected species of Oribatida (O) and Mesostigmata (M), dominance (*D*, %), constancy (*C*, %), indices in certain microhabitats of beech forest in the Wronie Reserve. Ad – adult, Juv – juveniles, Tot – totally. Species with mean density <20 are included.

Species		Beech litter	Moss on beech litter	Moss on beech stumps	Rotting wood	Marsh litter	Moss on beech trunks		Mean
							0.5 m a.l.[2]	2.0 m a.l.[2]	
Metabelba papilipes (Nicolet, 1855) (O)	Ad	10	6.5	7.0	4.8	0	7.2	6.8	
Damaeidae Berlese, 1896	Juv	5.5	1.8	3.5	0.5	0	0.2	3.0	
	Tot	15.5	8.3	10.5	5.3	0	7.4	9.8	8.12
	D	2.1	0.9	0.3	1.1	0	0.2	0.5	
	C	100	83	100	100	0	100	67	
Phthiracaridae (O)*	Tot	1.3	7.7	11.2	5.0	0.2	0.2	0	3.64
Phthiracaridae Perty, 1841	*D*	0.2	0.9	0.4	1.0	0.04	<0.01	0	
	C	33	100	100	100	17	17	0	
Paragamasus lapponicus (Trägårdh, 1910) (M)	Ad	0.5	4.8	1.0	0	0	0.2	0	
Parasitidae Oudemans, 1901	Juv	1.5	12.5	3.5	0	0	0	0.3	
	Tot	2.0	17.3	4.5	0	0	0.2	0.3	3.48
	D	1.43	24.6	4.2	0	0	0.4	0.7	
	C	33	100	100	0	0	17	17	
Hafenrefferia gilvipes (C.L. Koch, 1839) (O)	Ad	0	0	0.8	9.8	0	0.2	7.8	
Tenuialidae Jacot, 1929	Juv	0	0	0.2	0.8	0	0.5	2.3	
	Tot	0	0	1.0	10.7	0	0.7	10.2	3.21

	D	0	0	0.03	2.2	0	0.01	0.5	
	C	0	0	67	83	0	17	83	
Microtritia minima (Berlese, 1904) (O)*	Tot	0.8	0	6.3	0	0	1.0	12.8	3.00
Euphthiracaridae Jacot, 1930	*D*	0.1	0	0.2	0	0	0.02	0.7	
	C	33	0	67	0	0	50	67	
Scheloribates latipes (C.L. Koch, 1844) (O)	Ad	2.5	0.2	2.0	4.3	0.2	1.7	1.7	
Scheloribatidae Gradjean, 1933	Juv	1.8	0.5	0	4.2	0	0.5	0	
	Tot	4.3	0.8	2.0	8.5	0.2	2.2	1.7	2.89
	D	0.6	0.1	0.1	1.7	0.04	0.1	0.1	
	C	50	50	33	67	17	33	67	
Carabodes marginatus (Michael, 1884) (O)*	Tot	0.3	1.2	6.2	3.8	0	4.7	2.3	2.64
Carabodidae C.L. Koch, 1843	*D*	0.1	0.1	0.2	0.8	0	0.1	0.1	
	C	33	50	100	100	0	83	33	
Platyseius subglaber (Oudemans, 1902) (M)	Ad	0	0	0	0	14.5	0	0	
Blattisociidae, Garman, 1948	Juv	0	0	0	0	3.2	0	0	
	Tot	0	0	0	0	17.7	0	0	2.52
	D	0	0	0	0	19.6	0	0	
	C	0	0	0	0	100	0	0	
Suctobelba reticulata Moritz, 1970 (O)*	Tot	16.5	0	0	0	0	0	0	2.36
Suctobelbidae Jacot, 1938	*D*	2.2	0	0	0	0	0	0	
	C	67	0	0	0	0	0	0	
Oppiella subpectinata (Oudemans, 1900) (O)*	Tot	0	6.0	9.8	0	0	0	0.2	2.29
Oppiidae Grandjean, 1951	*D*	0	0.7	0.3	0	0	0	0.01	
	C	0	67	33	0	0	0	17	
Trachytes aegrota (C. L. Koch, 1841) (M)	Ad	2.7	0.7	1.8	0	0	2.4	1.7	
Trachytidae Trägårdh, 1938	Juv	1.5	0.7	1.5	0.2	0	0.8	0.8	
	Tot	4.2	1.4	3.3	0.2	0	3.2	2.5	2.10
	D	3.0	1.9	3.1	0.4	0	6.9	4.9	
	C	83	33	50	17	0	50	33	
Leptogamasus anoxygynellus (Micherdziński, 1969) (M)	Ad	6.7	0.3	0	0.7	0	0	0.3	
Parasitidae Oudemans, 1901	Juv	2.5	0.2	0.7	2.7	0	0	0	
	Tot	9.2	0.5	0.7	3.3	0	0	0.3	2.00
	D	6.6	0.7	0.6	8.2	0	0	0.7	
	C	83	33	17	83	0	0	17	
Poroliodes farinosus (C.L. Koch, 1840) (O)	Ad	0.2	0	1.3	3.7	0	1.2	0.5	
Neoliodidae Sellnick, 1928	Juv	0.2	0	1.5	1.8	0	2.2	0.8	
	Tot	0.4	0	2.8	5.5	0	3.4	1.3	1.90
	D	0.05	0	0.1	1.1	0	0.1	0.1	
	C	33	0	50	83	0	83	50	
Tritegeus bisulcatus Grandjean, 1953 (O)	Ad	0	9.7	0.2	0	0	0	0	
Cepheidae Berlese, 1896	Juv	0	2.0	0.3	0	0	0	0	
	Tot	0	11.7	0.5	0	0	0	0	1.74
	D	0	1.31	0.02	0	0	0	0	
	C	0	50	33	0	0	0	0	

Eupelops plicatus (C.L. Koch, 1836) (O)	Ad	3.7	2.8	0.2	0.8	0	0.5	0	
Phenopelopidae Petrunkevich, 1955	Juv	1.7	1.5	0.2	0.2	0	0.2	0.2	
	Tot	5.3	4.3	0.4	1.0	0	0.7	0.2	1.69
	D	0.7	0.5	0.01	0.2	0	0.01	0.01	
	C	100	87	33	67	0	50	17	
Ophidiotrichus tectus (Michael, 1884) (O)	Ad	0.8	2.2	1.3	1.5	0	0.7	4.8	
Oribatellidae Jacot, 1925	Juv	0	0	0	0	0	0	0	
	Tot	0.8	2.2	1.3	1.5	0	0.7	4.8	1.62
	D	0.1	0.2	0.04	0.3	0	0.01	0.3	
	C	33	67	33	33	0	33	67	
Damaeus riparius Nicolet, 1855 (O)	Ad	6.2	0.7	0.3	0	0.2	0	0.8	
Damaeidae Berlese, 1896	Juv	1.8	0.2	0	0	0	0.2	0.4	
	Tot	8.0	0.9	0.3	0	0.2	0.2	1.2	1.52
	D	1.1	0.1	0.01	0	0.04	<0.01	0.1	
	C	83	67	33	0	17	17	67	
Nothrus palustris C.L. Koch, 1839 (O)	Ad	0	1.0	1.5	0	0	0	0	
Nothridae Berlese, 1896	Juv	0.3	3.3	3.7	0	0	0	0	
	Tot	0.3	4.3	5.2	0	0	0	0	1.40
	D	0.1	0.5	0.2	0	0	0	0	
	C	17	33	50	0	0	0	0	
Autogneta longilamellata (Michael, 1885) (O)	Ad	0	1.0	5.8	2.2	0	0	0.7	
Autognetidae Grandjean, 1960	Juv	0	0	0	0	0	0	0	
	Tot	0	1.0	5.8	2.2	0	0	0.7	1.38
	D	0	0.1	0.2	0.4	0	0	0.04	
	C	0	50	50	50	0	0	17	
Malaconothrus monodactylus (Michael, 1888) (O)	Ad	0	4.8	0	0	0	0	0	
Malaconothridae Berlese, 1916	Juv	0	3.8	0	0	0	0	0	
	Tot	0	8.7	0	0	0	0	0	1.24
	D	0	1.0	0	0	0	0	0	
	C	0	17	0	0	0	0	0	
Ololaelaps placentula (Berlese, 1887) (M)	Ad	0	0.2	1.0	0.5	3.1	0.2	1.2	
Laelapidae Berlese, 1892	Juv	0	0	0.3	0.2	0.7	0	0.5	
	Tot	0	0.2	1.3	0.7	3.8	0.2	1.7	1.12
	D	0	0.2	1.2	1.6	4.3	0.4	3.3	
	C	0	17	50	17	17	17	50	
Vulgarogamasus kraepelini (Berlese, 1904) (M)	Ad	1.5	1.0	0	0	0	0	0	
Parasitidae Oudemans, 1901	Juv	1.3	3.5	0	0	0	0	0	
	Tot	2.8	4.5	0	0	0	0	0	1.10
	D	2.0	6.4	0	0	0	0	0	
	C	83	83	0	0	0	0	0	
Acrogalumna longipluma (Berlese, 1904) (O)	Ad	0	0.7	0.2	2.5	0	0.5	0.8	
Galumnidae Jacot, 1925	Juv	0	0	0	1.3	0	0.8	0	
	Tot	0	0.7	0.2	3.8	0	1.3	0.8	0.98
	D	0	0.1	0.01	0.8	0	0.03	0.04	

	C	0	33	17	83	0	50	50	
Holoparasitus excipuliger (Berlese, 1906) (M)	Ad	0	0	0.9	0.8	0	2.8	0.2	
Parasitidae Oudemans, 1901	Juv	0	0	0.8	0	0	0.5	0.2	
	Tot	0	0	1.7	0.8	0	3.3	0.4	0.88
	D	0	0	1.6	2.1	0	7.3	0.7	
	C	0	0	67	33	0	100	33	
Microppia minus (Paoli, 1908) (O)*	Tot	3.0	1.3	1.8	0	0	0	0	0.88
Oppiidae Grandjean, 1951	*D*	0.41	1.2	0.1	0	0	0	0	
	C	33	50	17	0	0	0	0	
Carabodes ornatus Storkan, 1925 (O)*	Tot	1.0	0	2.5	0	0	0.5	2.0	0.86
Carabodidae C.L. Koch, 1843	*D*	0.14	0	0.1	0	0	0.01	0.11	
	C	33	0	83	0	0	17	33	
Liacarus coracinus (C.L. Koch, 1841) (O)*	Tot	1.2	1.0	3.3	0	0	0.3	0	0.83
Liacaridae Sellnick, 1928	*D*	0.16	0.11	0.11	0	0	0.01	0	
	C	67	50	67	0	0	33	0	
Oodinychus ovalis (C. L. Koch, 1839) (M)	Ad	0	0	0	1.8	0	0	2.5	
Trematuridae Berlese, 1917	Juv	0	0	0	1.2	0	0	0.2	
	Tot	0	0	0	3.0	0	0	2.7	0.81
	D	0	0	0	7.4	0	0	5.2	
	C	0	0	0	33	0	0	33	
Celaenopsis badius (C. L. Koch, 1839) (M)	Ad	0	0	0	0	0	4.2	0.2	
Celaenopsidae Berlese, 1892	Juv	0	0	0	0.3	0	0.8	0	
	Tot	0	0	0	0.3	0	5.0	0.2	0.78
	D	0	0	0	0.8	0	11	0.33	
	C	0	0	0	33	0	67	17	
Amblyseius sp. (M)	Ad	0	0.2	2.8	0	0	0.3	0	
Phytoseiidae Berlese, 1916	Juv	0	0	2.7	0.2	0	0	0	
	Tot	0	0.2	5.5	0.2	0	0.3	0	0.74
	D	0	0.2	4.2	0.4	0	0.7	0	
	C	0	17	83	17	0	17	0	
Damaeus auritius C.L. Koch, 1835 (O)	Ad	2.5	0	0	0.5	0	0.5	0.2	
Damaeidae Berlese, 1896	Juv	0.7	0	0	0.2	0	0.3	0	
	Tot	3.2	0	0	0.7	0	0.8	0.2	0.70
	D	0.43	0	0	0.1	0	0.02	0.01	
	C	100	0	0	17	0	50	17	
Astegistes pilosus (C.L. Koch, 1840) (O)*	Tot	0	0	0	0	4.5	0	0	0.64
Astegistidae Balogh, 196l	*D*	0	0	0	0	0.1	0	0	
	C	0	0	0	0	83	0	0	
Poecilochthonius italicus (Berlese, 1910) (O)*	Tot	4.2	0	0	0	0	0	0	0.6
Brachychthoniidae Thor, 1934	*D*	0.6	0	0	0	0	0	0	
	C	50	0	0	0	0	0	0	
Camisia spinifer (C.L. Koch, 1835) (O)	Ad	0	0.5	0.8	0	0	0.3	0.3	
Camisiidae Oudemans, 1900	Juv	0.2	0.2	0.2	0	0	1.7	0	
	Tot	0.2	0.7	1.0	0	0	2.0	0.3	0.60
	D	0.02	0.07	0.03	0	0	0.04	0.02	

	C	17	33	50	0	0	83	33	
Oribatella calcarata (C.L. Koch, 1835) (O)*	Tot	0	0	1.0	1.7	0	0	1.3	0.57
Oribatellidae Jacot, 1925	*D*	0	0	0.03	0.3	0	0	0.07	
	C	0	0	50	63	0	0	83	
Steganacarus magnus (Nicolet, 1855) (O)*	Tot	1.8	1.3	0.2	0	0	0.5	0	0.55
Phthiracaridae Perty, 1841	*D*	0.3	0.2	0.01	0	0	0.01	0	
	C	33	83	17	0	0	33	0	
Cepheus dentatus (Michael, 1888) (O)	Ad	0	0	0.8	1.3	0	0.3	0.2	
Cepheidae Berlese, 1896	Juv	0	0	0.5	0.2	0	0	0	
	Tot	0	0.5	1.3	1.5	0	0.3	0.2	0.55
	D	0	0.1	0.04	0.3	0	0.01	0.01	
	C	0	17	67	33	0	33	17	
Zetorchestes falzonii Coggi, 1898 (O)	Ad	0.3	0.8	0	0	1.3	0	0	
Zetorchestidae Michael, 1898	Juv	0.5	0	0	0	0	0	0	
	Tot	0.8	0.8	0	0	1.3	0	0	0.43
	D	0.11	0.1	0	0	0.3	0	0	
	C	17	50	0	0	17	0	0	
Hypoaspis miles (Berlese, 1892) (M)	Ad	0	0	0	0	0	0	0	
Laelapidae Berlese, 1892	Juv	0	0	0	3.0	0	0	0	
	Tot	0	0	0	3.0	0	0	0	0.43
	D	0	0	0	7.4	0	0	0	
	C	0	0	0	50	0	0	0	
Cosmolaelaps vacua (Michael, 1891) (M)	Ad	0	0	0	0.3	0	0	0	
Laelapidae Berlese, 1892	Juv	0	0	0	1.7	0	0	0	
	Tot	0	0	0	2.0	1.0	0	0	0.43
	D	0	0	0	4.9	1.1	0	0	
	C	0	0	0	33	17	0	0	
Dinychus perforatus Kramer, 1886 (M)	Ad	0.2	0	0	0.5	0	0	0	
Dinychidae Berlese, 1916	Juv	0	0	0	2.2	0	0	0	
	Tot	0.2	0	0	2.7	0	0	0	0.43
	D	0.1	0	0	6.6	0	0	0	
	C	17	0	0	50	0	0	0	
Uroobovella obovata (Canestrini & Berlese,	Ad	0	0	0	0.2	0	0	2.3	
1884) (M)									
Urodinychidae Berlese, 1917	Juv	0	0	0	0.2	0	0	0	
	Tot	0	0	0	0.4	0	0	2.3	0.38
	D	0	0	0	0.8	0	0	4.6	
	C	0	0	0	33	0	0	50	
Multioppia glabra (Mihelcic, 1955) (O)*	Tot	1.3	1.3	0	0	0	0	0	0.38
Oppiidae Grandjean, 1951	*D*	0.2	0.2	0	0	0	0	0	
	C	33	33	0	0	0	0	0	
Dendrolaelaps rectus Karg, 1962 (M)	Ad	0	0	0	1.2	0	0	0	
Digamasellidae Evans, 1957	Juv	0	0	0	1.5	0	0	0	
	Tot	0	0	0	2.7	0	0	0	0.38
	D	0	0	0	6.6	0	0	0	

	C	0	0	0	83	0	0	0	
Cultroribula bicultrata (Berlese, 1905) (O)*	Tot	0.3	1.0	0.5	0.3	0	0	0.2	0.33
Astegistidae Balogh, 196l	*D*	0.05	0.11	0.02	0.07	0	0	0.01	
	C	33	67	33	17	0	0	17	
Pergamasus brevicornis Berlese, 1903 (M)	Ad	0	0	0.3	0	0	0	0.2	
Parasitidae Oudemans, 1901	Juv	0.3	0	1.0	0	0	0.3	0.2	
	Tot	0.3	0	1.3	0	0	0.3	0.3	0.33
	D	0.2	0	1.2	0	0	0.6	0.7	
	C	33	0	67	0	0	33	17	
Ceratoppia sexpilosa Willmann, 1938 (O)	Ad	0.7	0.7	0.2	0	0	0.2	0	
Peloppiidae Balogh, 1943	Juv	0	0.5	0	0	0	0	0	
	Tot	0.7	1.2	0.2	0	0	0.2	0	0.31
	D	0.1	0.1	0.01	0	0	<0.01	0	
	C	67	33	17	0	0	17	0	
Arctoseius cetratus (Sellnick, 1940) (M)	Ad	1.5	0	0	0	0.3	0	0	
Ascidae Oudemans, 1905	Juv	0.3	0	0	0	0	0	0	
	Tot	1.8	0	0	0	0.3	0	0	0.31
	D	1.3	0	0	0	0.4	0	0	
	C	50	0	0	0	33	0	0	
Stegatacarus striculus (C.L. Koch, 1835) (O)*	Tot	1.3	0	0	0	0	0.7	0	0.29
Phthiracaridae Perty, 1841	*D*	0.18	0	0	0	0	0.01	0	
	C	17	0	0	0	0	17	0	
Veigaia cerva (Kramer, 1876) (M)	Ad	0.2	0.2	0	0.2	0	0	0	
Veigaiaidae Oudemans, 1939	Juv	0	0.5	03	0.5	0	0.2	0	
	Tot	0.2	0.7	0.3	0.7	0	0.2	0	0.29
	D	0.1	1.0	0.3	1.6	0	0.4	0	
	C	17	33	33	17	0	17	0	
Dendrolaelaps arvicolus (Leitner, 1949) (M)	Ad	0	0	0	1.5	0	0	0	
Digamasellidae Evans, 1957	Juv	0	0	0	0.5	0	0	0	
	Tot	0	0	0	2.0	0	0	0	0.29
	D	0	0	0	4.9	0	0	0	
	C	0	0	0	67	0	0	0	
Pilogalumna crasiclava (Berlese, 1914) (O)	Ad	0	0	0	0	0	0.2	0	
Galumnidae Jacot, 1925	Juv	0	0	0.2	0	0	0	0	
	Tot	1.2	0.3	0.2	0	0	0.2	0	0.26
	D	0.16	0.04	0.01	0	0	<0.01	0	
	C	50	33	33	0	0	17	0	
Ceratozetes gracilis (Michael, 1884) (O)*	Tot	0	1.5	0.3	0	0	0	0	0.26
Ceratozetidae Jacot, 1925	*D*	0	0.17	0.01	0	0	0	0	
	C	0	33	17	0	0	0	0	
Arctoseius insularis (Willmann, 1952) (M)	Ad	0	0	0	1.0	0	0	0	
Ascidae Oudemans, 1905	Juv	0	0	0	0.2	0	0	0	
	Tot	0	0	0	1.2	0.5	0	0	0.24
	D	0	0	0	2.9	0.6	0	0	
	C	0	0	0	67	17	0	0	

Micreremaeus brevipes (Michael, 1888) (O)*	Tot	0.2	1.0	0.3	0	0	0	0.2	0.24
Micreremidae Grandjean, 1954	*D*	0.02	0.11	0.01	0	0	0	0.01	
	C	17	50	33	0	0	0	17	
Dendrolaelaps latus Hirschmann, 1960 (M)	Ad	0	0	0	0.7	0	0	0	
Digamasellidae Evans, 1957	Juv	0	0	0	0.8	0	0	0.2	
	Tot	0	0	0	1.5	0	0	0.2	0.24
	D	0	0	0	3.7	0	0	0.3	
	C	0	0	0	50	0	0	17	
Dendrolaelaps disetosimilis Hirschmann, 1960 (M)*	Tot	0	0	0	1.5	0	0	0	0.21
Digamasellidae Evans, 1957	*D*	0	0	0	3.7	0	0	0	
	C	0	0	0	33	0	0	0	
Gaeolaelaps praesternalis (Willman,1949) (M)*	Tot	0.2	0	1.2	0	0	0	0	0.19
Laelapidae Berlese, 1892	*D*	0.1	0	1.1	0	0	0	0	
	C	17	0	67	0	0	0	0	
Scheloribates pallidulus (C.L. Koch, 1841) (O)*	Tot	0	0	1.3	0	0	0	0	0.19
Scheloribatidae Gradjean, 1933	*D*	0	0	0.04	0	0	0	0	
	C	0	0	17	0	0	0	0	
Macrocheles sp. (M)	Ad	0	0.1	0.2	0	0	0	0	
Macrochelidae Vitzthum, 1930	Juv	0	0.2	0	0.2	0	0.2	0	
	Tot	0.5	0.3	0.2	0.2	0	0.2	0	0.19
	D	0.4	0.5	0.2	0.4	0	0.4	0	
	C	33	17	17	17	0	17	0	
Paragamasus runciger (Berlese, 1903) (M)	Ad	0	0	0	0.1	0	0	0	
Parasitidae Oudemans, 1901	Juv	0	0	0	1.2	0	0	0	
	Tot	0	0	0	1.3	0	0	0	0.19
	D	0	0	0	3.3	0	0	0	
	C	0	0	0	50	0	0	0	
Urodiaspis pannonica Willmann, 1951 (M)	Ad	0.8	0	0	0	0	0	0	
Dinychidae Berlese, 1916	Juv	0.5	0	0	0	0	0	0	
	Tot	1.3	0	0	0	0	0	0	0.19
	D	1.0	0	0	0	0	0	0	
	C	50	0	0	0	0	0	0	
Rhysotritia duplicata (Grandjean, 1953) (O)*	Tot	1.2	0	0	0	0	0	0	0.17
Euphthiracaridae Jacot, 1930	*D*	0.16	0	0	0	0	0	0	
	C	17	0	0	0	0	0	0	
Liebstadia humerata Sellnick, 1928 (O)	Ad	0.7	0	0	0	0	0.2	0	
Scheloribatidae Gradjean, 1933	Juv	0.3	0	0	0	0	0	0	
	Tot	1.0	0	0	0	0	0.2	0	0.17
	D	0.14	0	0	0	0	<0.01	0	
	C	50	0	0	0	0	17	0	
Oribella castanea Willmann, 1931 (O)*	Tot	0	0	0	1.2	0	0	0	0.17
Thyrisomidae Grandjean, 1954	*D*	0	0	0	0.2	0	0	0	
	C	0	0	0	67	0	0	0	
Iphidozercon gibbus (Berlese, 1903) (M)	Ad	0	0	0	0	1.5	0	0	

Ascidae Oudemans, 1905	Juv	0	0	0	0	0.2	0	0	
	Tot	0	0	0	0	1.7	0	0	0.17
	D	0	0	0	0	1.3	0	0	
	C	0	0	0	0	50	0	0	
Paragamasus runcatellus Berlese, 1903 (M)	Ad	0.8	0.3	0	0	0	0	0	
Parasitidae Oudemans, 1901	Juv	0	0	0	0	0	0	0	
	Tot	0.8	0.3	0	0	0	0	0	0.17
	D	0.6	0.5	0	0	0	0	0	
	C	33	17	0	0	0	0	0	
Pergamasus persissus (Michердziński, 1969) (M)*	Tot	1.2	0	0	0	0	0	0	0.17
Parasitidae Oudemans, 1901	*D*	0.8	0	0	0	0	0	0	
	C	17	0	0	0	0	0	0	
Leptogamasus suecicus Trägårdh, 1936 (M)*	Tot	1.2	0	0	0	0	0	0	0.17
Parasitidae Oudemans, 1901	*D*	0.8	0	0	0	0	0	0	
	C	17	0	0	0	0	0	0	
Liacarus nitens (Gervais, 1844) (O)*	Tot	0.8	0	0.2	0	0	0	0	0.14
Liacaridae Sellnick, I92B	*D*	0.11	0	0.01	0	0	0	0	
	C	33	0	17	0	0	0	0	
Oribatella sexdentata Berlese, 1916 (O)*	Tot	0	0	1.0	0	0	0	0	0.14
Oribatellidae Jacot, 1925	*D*	0	0	0.03	0	0	0	0	
	C	0	0	33	0	0	0	0	
Pergamasus viator Halaškova, 1959 (M)	Ad	0	0.3	0	0	0	0	0	
Parasitidae Oudemans, 1901	Juv	0	0.5	0	0	0	0	0	
	Tot	0.2	0.8	0	0	0	0	0	0.14
	D	0.1	1.2	0	0	0	0	0	
	C	17	50	0	0	0	0	0	
Uropoda hamulifera Michael, 1894 (M)	Ad	0	0	0	0	0	0	0.5	
Uropodidae Kramer, 1881	Juv	0	0	0	0.3	0	0	0.2	
	Tot	0	0	0	0.3	0	0	0.7	0.14
	D	0	0	0	0.8	0	0	1.3	
	C	0	0	0	33	0	0	33	
Dendrolaelaps zwoelferi Hirschmann, 1960 (M)	Ad	0	0	0	0	0	0	0	
Digamasellidae Evans, 1957	Juv	0	0	0	1.0	0	0	0	
	Tot	0	0	0	1.0	0	0	0	0.14
	D	0	0	0	2.5	0	0	0	
	C	0	0	0	50	0	0	0	
Xenillus tegeocranus (Hermann, 1804) (O)*	Tot	0.2	0.2	0	0	0	0.5	0	0.12
Liacaridae Sellnick, 1928	*D*	0.02	0.02	0	0	0	0.01	0	
	C	17	17	0	0	0	50	0	
Diapterobates humeralis (Hermann, 1804) (O)*	Tot	0	0	0	0	0.8	0	0	0.12
Ceratozetidae Jacot, 1925	*D*	0	0	0	0	0.2	0	0	
	C	0	0	0	0	50	0	0	
Poecilochthonius spiciger (Berlese, 1910) (O)*	Tot	0	0	0	0.8	0	0	0	0.12
Brachychthoniidae Thor, 1934	*D*	0	0	0	0.2	0	0	0	

	C	0	0	0	33	0	0	0	
Geholaspis longispinosus (Kramer, 1876) (M)	Ad	0	0	0	0	0	0	0.3	
Macrochelidae Vitzthum, 1930	Juv	0	0	0	0	0	0	0.2	
	Tot	0	0.2	0	0.2	0	0	0.5	0.12
	D	0	0.2	0	0.4	0	0	1.0	
	C	0	17	0	17	0	0	33	
Lasioseius sp. (M)*	Tot	0	0	0	0	0.7	0	0	0.10
Ascidae Oudemans, 1905	*D*	0	0	0	0	0.7	0	0	
	C	0	0	0	0	33	0	0	
Gamasellodes bicolor (Berlese, 1918) (M)*	Tot	0.3	0	0.3	0	0	0	0	0.10
Ascidae Oudemans, 1905	*D*	0.2	0	0.3	0	0	0	0	
	C	17	0	33	0	0	0	0	
Parasitus sp. (M)	Ad	0	0	0	0	0	0	0	
Parasitidae Oudemans, 1901	Juv	0	0	0	0	0.2	0	0.5	
	Tot	0	0	0	0	0.2	0	0.5	0.10
	D	0	0	0	0	0.2	0	1.0	
	C	0	0	0	0	17	0	17	
Dinychus sp. (M)	Ad	0	0	0	0	0	0	0.2	
Dinychidae Berlese, 1916	Juv	0	0	0.2	0	0	0	0.2	
	Tot	0	0	0.2	0	0	0	0.4	0.07
	D	0	0	0.2	0	0	0	0.7	
	C	0	0	17	0	0	0	33	
Neojordensia levis Oudemans & Voigts, 1904 (M)*	Tot	0	0	0	0	0.5	0	0	0.07
Ascidae Oudemans, 1905	*D*	0	0	0	0	1.5	0	0	
	C	0	0	0	0	33	0	0	
Prozercon traegardhi (Halbert, 1923) (M)*	Tot	0.2	0	0.3	0	0	0	0	0.07
Zerconidae Berlese, 1892	*D*	0.1	0	0.3	0	0	0	0	
	C	17	0	17	0	0	0	0	
Paragamasus misellus Berlese, 1903 (M)	Ad	0	0	0	0.3	0	0	0	
Parasitidae Oudemans, 1901	Juv	0	0	0	0.2	0	0	0	
	Tot	0	0	0	0.5	0	0	0	0.07
	D	0	0	0	1.2	0	0	0	
	C	0	0	0	17	0	0	0	
Steganacarus carinatus (C.L. Koch, 1841) (O)*	Tot	0.3	0	0	0	0	0	0	0.05
Phthiracaridae Perty, 1841	*D*	0.05	0	0	0	0	0	0	
	C	17	0	0	0	0	0	0	
Cymbaeremaeus cymba (Nicolet, 1855) (O)*	Tot	0	0	0.3	0	0	0	0	0.05
Cymbaeremaeidae Sellnick, 1928	*D*	0	0	0.01	0	0	0	0	
	C	0	0	17	0	0	0	0	
Parasitus lunulatus (Müller, 1859) (M)	Ad	0	0	0	0	0	0	0	
Parasitidae Oudemans, 1901	Juv	0	0	0	0	0	0.2	0	
	Tot	0	0	0.2	0	0	0.2	0	0.05
	D	0	0	0.2	0	0	0.4	0	
	C	0	0	17	0	0	17	0	

Proctolaelaps pygmaeus (Müller, 1860) (M)*	Tot	0	0	0	0.2	0	0.2	0	0.05
Melicharidae Hirschmann, 1962	*D*	0	0	0	0.4	0	0.4	0	
	C	0	0	0	17	0	17	0	
Anthoseius verrucosus Wainstein, 1972 (M)*	Tot	0	0	0	0.2	0.2	0	0.2	0.05
Phytoseiidae Berlese, 1916	*D*	0	0	0	0.4	0.4	0	0.3	
	C	0	0	0	17	17	0	17	
Zerconopsis remiger (Kramer, 1876) (M)*	Tot	0	0	0.2	0.2	0	0	0	0.05
Ascidae Oudemans, 1905	*D*	0	0	0.2	0.4	0	0	0	
	C	0	0	17	17	0	0	0	
Dendrolaelaps ulmi Hirschmann, 1960 (M)*	Tot	0	0	0	0.3	0	0	0	0.05
Digamasellidae Evans, 1957	*D*	0	0	0	0.8	0	0	0	
	C	0	0	0	33	0	0	0	
Pergamasus quisquiliarum (Canestrini & Canestrini, 1882) (M)	Ad	0.2	0	0	0	0	0	0	
Parasitidae Oudemans, 1901	Juv	0.2	0	0	0	0	0	0	
	Tot	0.4	0	0	0	0	0	0	0.05
	D	0.2	0	0	0	0	0	0	
	C	17	0	0	0	0	0	0	
Olodiscus minima (Kramer, 1882) (M)*	Tot	0.3	0	0	0	0	0	0	0.05
Uropodidae Kramer, 1881	*D*	0.2	0	0	0	0	0	0	
	C	17	0	0	0	0	0	0	
Leitneria granulata (Halbert, 1923) (M)*	Tot	0.3	0	0	0	0	0	0	0.05
Halolaelapidae Karg, 1965	*D*	0.2	0	0	0	0	0	0	
	C	17	0	0	0	0	0	0	
Gamasida 1 (M)*	Tot	0	0	0	0	0	0.2	0	0.03
	D	0	0	0	0	0	0.4	0	
	C	0	0	0	0	0	17	0	
Eupelops subuliger (Berlese, 1916) (O)*	Tot	0	0	0	0	0	0.2	0	0.02
Phenopelopidae Petrunkevich, 1955	*D*	0	0	0	0	0	<0.01	0	
	C	0	0	0	0	0	17	0	
Eniochthonius minutissimus (Berlese, 1903) (O)*	Tot	0	0	0	0	0.2	0	0	0.02
Eniochthoniidae Grandjean, 1947	*D*	0	0	0	0	0.04	0	0	
	C	0	0	0	0	17	0	0	
Amblyseius rademacheri Dosse, 1958 (M)*	Tot	0	0	0	0	0	0.2	0	0.02
Phytoseiidae Berlese, 1916	*D*	0	0	0	0	0	0.4	0	
	C	0	0	0	0	0	17	0	
Asca nova Willmann, 1939 (M)*	Tot	0	0.2	0	0	0	0	0	0.02
Ascidae Oudemans, 1905	*D*	0	0.2	0	0	0	0	0	
	C	0	17	0	0	0	0	0	
Proctolaelaps sp. (M)	Ad	0	0	0	0	0	0	0	
Melicharidae Hirschmann, 1962	Juv	0	0	0	0	0	0	0.2	
	Tot	0	0	0	0	0	0	0.2	0.02
	D	0	0	0	0	0	0	0.3	
	C	0	0	0	0	0	0	17	

Sejus togatus C. L. Koch, 1836 (M)*	Tot	0	0	0	0	0	0	0.2	0.02
Sejidae Berlese, 1885	*D*	0	0	0	0	0	0	0.3	
	C	0	0	0	0	0	0	17	
Uropoda sp. (M)	Ad	0	0	0	0	0	0	0	
Uropodidae Kramer, 1881	Juv	0	0	0	0	0	0	0.2	
	Tot	0	0	0	0	0	0	0.2	0.02
	D	0	0	0	0	0	0	0.3	
	C	0	0	0	0	0	0	17	
Zerconopsis sp. (M)	Ad	0	0	0	0	0	0	0	
Ascidae Oudemans, 1905	Juv	0	0	0	0	0	0	0.2	
	Tot	0	0	0	0	0	0	0.2	0.02
	D	0	0	0	0	0	0	0.3	
	C	0	0	0	0	0	0	17	
Eviphis ostrinus (C.L. Koch, 1836) (M)	Ad	0	0	0	0	0	0	0	
Eviphididae Berlese, 1913	Juv	0	0	0.2	0	0	0	0	
	Tot	0	0	0.2	0	0	0	0	0.02
	D	0	0	0.2	0	0	0	0	
	C	0	0	17	0	0	0	0	
Zerconopsis apodius Karg, 1969 (M)*	Tot	0	0	0	0.2	0	0	0	0.02
Ascidae Oudemans, 1905	*D*	0	0	0	0.4	0	0	0	
	C	0	0	0	17	0	0	0	
Hypoaspis sp. (M)	Ad	0	0	0	0	0	0	0	
Laelapidae Berlese, 1892	Juv	0	0	0	0.2	0	0	0	
	Tot	0	0	0	0.2	0	0	0	0.02
	D	0	0	0	0.4	0	0	0	
	C	17	0	0	0	0	0	0	
Geholaspis mandibularis (Berlese, 1904) (M)	Ad	0	0	0	0	0	0	0	
Macrochelidae Vitzthum, 1930	Juv	0.2	0	0	0	0	0	0	
	Tot	0.2	0	0	0	0	0	0	0.02
	D	0.1	0	0	0	0	0	0	
	C	17	0	0	0	0	0	0	
Pachylaelaps sp. (M)*	Tot	0.2	0	0	0	0	0	0	0.02
Pachylaelapidae Berlese, 1913	*D*	0.1	0	0	0	0	0	0	
	C	17	0	0	0	0	0	0	

* species without juveniles stages

[1] – Mean density [individuals in 500 cm^3 ± SD (standard deviation)]; [2] – a. l. – above the litter

Table S3. Domination structure of Mesostigmata communities in chosen microhabitats of beech forest in Wronie Reserve. Domination index of species is given in brackets.

	Beach litter	Moss on beach litter	Moss on beach stumps	Rotting wood	Marsh litter	0.5 m a.l.	2.0 m a.l.
**Superdominants**	*Zercon peltatus* (47.79)	*Veigaia nemorensis* (49.17)	*Zercon peltatus* (68.16)		*Platyseius italicus* (46.67)	*Zercon peltatus* (63.14)	*Zercon peltatus* (62.87)
**Eudominants**	*Zercon gurensis* (23.54)	*Paragamasus lapponicus* (24.59)			*Cheiroseius* sp. (23.52)		
**Dominants**		*Zercon gurensis* (13.24)		*Zercon peltatus* (18.03)	*Platyseius subglaber* (19.63)	*Veigaia nemorensis* (12.04)<br>*Celaenopsis badius* (10.95)	*Veigaia nemorensis* (10.42)
**Subdominants**	*Veigaia nemorensis* (8.72)<br>*Pergamasus anoxygynellus* (6.57)	*Vulgarogamasus kraepelini* (6.38)	*Veigaia nemorensis* (9.27)	*Pergamasus anoxygynellus* (8.20)<br>*Trichouropoda ovalis* (7.38)<br>*Hypoaspis milles* (7.38)<br>*Dendrolaelaps rectus* (6.56)<br>*Dinychus perforatus* (6.56)<br>*Veigaia nemorensis* (6.15)		*Holoparasitus excipuliger* (7.30)<br>*Trachytes aegrota* (6.93)<br>*Zercon gurensis* (6.20)	*Trichouropoda ovalis* (5.21)
**Recedents**	*Trachytes aegrota* (2.99)<br>*Vulgarogamasus kraepelini* (2.03)<br>*Paragamasus lapponicus* (1.43)<br>*Arctoseius cetratus* (1.31)	*Trachytes aegrota* (1.89)<br>*Pergamasus viator* (1.18)	*Paragamasus lapponicus*<br>*Amblyseius* sp. (4.17)<br>*Zercon gurensis* (3.71)<br>*Trachytes aegrota* (3.09)<br>*Holoparasitus excipuliger* (1.55)<br>*Ololaelaps placentula* (1.24)<br>*Pergemasus brevicornis* (1.24)<br>*Hypoaspis praesternalis* (1.08)	*Dendrolaelaps arvicolus* (4.92)<br>*Hypoaspis vacua* (4.92)<br>*Dendrolaelaps latus* (3.69)<br>*Dendrolaelaps disetosimilis* (3.69)<br>*Pergamasus runciger* (3.28)<br>*Arctoseius insularis* (2.87)<br>*Dendrolaelaps zwolferi*<br>*Holoparasitus excipuliger* (2.05)<br>*Ololaelaps placentula* (1.64)<br>*Veigaia cerva* (1.64)<br>*Zercon gurensis* (1.23)<br>*Pergamasus misellus* (1.23)	*Ololaelaps placentula* (4.26)<br>*Neojordensia laevis* (1.48)<br>*Iphidozercon gibbus* (1.32)<br>*Hypoaspis vacua* (1.11)		*Trachytes aegrota* (4.89)<br>*Urobovella obovata* (4.56)<br>*Ololaelaps placentula* (3.26)<br>*Uropoda hamulifera* (1.30)

**Subrecedents**	*Uropdiaspis pannonica* (0.96)<br>*Pergamasus persissus* (0.84)<br>*Pergamasus suecicus* (0.84)<br>*Paragamasus runcatellus* (0.60)<br>*Macrocheles* sp. (0.36)<br>*Pergemasus brevicornis* (0.24)<br>*Gamasellodes bicolor* (0.24)<br>*Pergamasus quisquiliarum* (0.24)<br>*Uropoda minima* (0.24)<br>*Leitneria granulata* (0.24)<br>*Veigaia cerva* (0.12)<br>*Pergamasus viator* (0.12)<br>*Hypoaspis praesternalis* (0.12)<br>*Prozercon traegardhi* (0.12)<br>*Dinychus perforatus* (0.12)<br>*Geholaspis mandibularis* (0.12)<br>*Pachylaelaps* sp. (0.12)	*Veigaia cerva* (0.95)<br>*Pergamasus anoxygynellus* (0.71)<br>*Macrocheles* sp. (0.47)<br>*Paragamasus runcatellus* (0.47)<br>*Ololaelaps placentula* (0.24)<br>*Amblyseius* sp. (0.24)<br>*Asca nova* (0.24)<br>*Geholaspis longispinosus* (0.24)	*Pergamasus anoxygynellus* (0.62)<br>*Veigaia cerva* (0.31)<br>*Gamasellodes bicolor* (0.31)<br>*Prozercon traegardhi* (0.31)<br>*Parasitus lunulatus* (0.15)<br>*Macrocheles* sp. (0.15)<br>*Dinychus* sp. (0.15)<br>*Zerconopsis remiger* (0.15)<br>*Eviphis ostrinus* (0.15)	*Urobovella obovata* (0.82)<br>*Uropoda hamulifera* (0.82)<br>*Dendrolaelaps ulmi* (0.82)<br>*Celaenopsis badius* (0.81)<br>*Trachytes aegrota* (0.41)<br>*Amblyseius* sp. (0.41)<br>*Macrocheles* sp. (0.41)<br>*Proctolaelaps pygmaeus* (0.41)<br>*Geholaspis longispinosus* (0.41)<br>*Anthoseius verrucosus* (0.41)<br>*Zerconopsis remiger* (0.41)<br>*Zerconopsis apodius* (0.41)<br>*Hypoaspis* sp. (0.41)	*Lasioseius* sp. (0.74)<br>*Arctoseius insularis* (0.56)<br>*Anthoseius verrucosus* (0.37)<br>*Arctoseius cetratus* (0.37)<br>*Parasitus* sp. (0.19)	*Pergemasus brevicornis* (0.73)<br>*Amblyseius* sp. (0.73)<br>*Gamasida* 4 (0.36)<br>*Ololaelaps placentula* (0.36)<br>*Paragamasus lapponicus* (0.36)<br>*Parasitus lunulatus* (0.36)<br>*Veigaia cerva* (0.36)<br>*Amblyseius rademacheri* (0.36)<br>*Macrocheles* sp. (0.36)<br>*Proctolaelaps pygmaeus* (0.36)	*Geholaspis longispinosus* (0.98)<br>*Parasitus* sp. (0.98)<br>*Holoparasitus excipuliger* (0.65)<br>*Paragamasus lapponicus* (0.65)<br>*Pergemasus brevicornis* (0.65)<br>*Pergamasus anoxygynellus* (0.65)<br>*Dinychus* sp. (0.65)<br>*Celaenopsis badius* (0.33)<br>*Zercon gurensis* (0.33)<br>*Anthoseius verrucosus* (0.33)<br>*Dendrolaelaps latus* (0.33)<br>*Proctolaelaps* sp. (0.33)<br>*Sejus togatus* (0.33)<br>*Uropoda* sp. (0.33)<br>*Zerconopsis* sp. (0.33)
